# Possible health impacts of Bt toxins and residues from spraying with complementary herbicides in genetically engineered soybeans and risk assessment as performed by the European Food Safety Authority EFSA

**DOI:** 10.1186/s12302-016-0099-0

**Published:** 2017-01-11

**Authors:** Christoph Then, Andreas Bauer-Panskus

**Affiliations:** Testbiotech, Institute for Independent Impact Assessment in Biotechnology, Frohschammerstr. 14, 80807 Munich, Germany

**Keywords:** Genetically engineered plants, Bt toxins, Herbicide residues, Health effects, Risk assessment

## Abstract

**Background:**

MON89788 was the first genetically engineered soybean worldwide to express a Bt toxin. Under the brand name *Intacta,* Monsanto subsequently engineered a stacked trait soybean using MON89788 and MON87701—this stacked soybean expresses an insecticidal toxin and is, in addition, tolerant to glyphosate. After undergoing risk assessment by the European Food Safety Authority (EFSA), the stacked event was authorised for import into the EU in June 2012, including for use in food and feed. This review discusses the health risks associated with Bt toxins present in these genetically engineered plants and the residues left from spraying with the complementary herbicide.

**Results:**

We have compared the opinion published by EFSA [1] with findings from other publications in the scientific literature. It is evident that there are several issues that EFSA did not consider in detail and which will need further assessment: (1) There are potential combinatorial effects between plant components and other impact factors that might enhance toxicity. (2) It is known that Bt toxins have immunogenic properties; since soybeans naturally contain many allergens, these immunogenic properties raise specific questions. (3) Fully evaluated and reliable protocols for measuring the Bt concentration in the plants are needed, in addition to a comprehensive set of data on gene expression under varying environmental conditions. (4) Specific attention should be paid to the herbicide residues and their interaction with Bt toxins.

**Conclusions:**

The case of the *Intacta* soybeans highlights several regulatory problems with Bt soybean plants in the EU. Moreover, many of the issues raised also concern other genetically engineered plants that express insecticidal proteins, or are engineered to be resistant to herbicides, or have those two types of traits combined in stacked events. It remains a matter of debate whether the standards currently applied by the risk assessor, EFSA, and the risk manager, the EU Commission, meet the standards for risk analysis defined in EU regulations such as 1829/2003 and Directive 2001/18. While this publication cannot provide a final conclusion, it allows the development of some robust hypotheses that should be investigated further before such plants can be considered to be safe for health and the environment. In general, the concept of comparative risk assessment needs some major revision. Priority should be given to developing more targeted approaches. As shown in the case of *Intacta,* these approaches should include: (i) systematic investigation of interactions between the plant genome and environmental stressors as well as their impact on gene expression and plant composition; (ii) detailed investigations of the toxicity of Bt toxins; (iii) assessment of combinatorial effects taking into account long-term effects and the residues from spraying with complementary herbicides; (iv) investigation into the impact on the immune and hormonal systems and (v) investigation of the impact on the intestinal microbiome after consumption. Further and in general, stacked events displaying a high degree of complexity due to possible interactions should not undergo a lower level of risk assessment than the parental plants.

## Background

After undergoing risk assessment by EFSA, the genetically engineered stacked soybean MON87701 × MON89788, produced by Monsanto and sold under the brand name *Intacta,* was authorised for import and use in food and feed in the EU [1]. The soybeans combine the expression of an insecticidal Bt toxin, Cry1Ac, present in the parental event MON87701, with herbicide resistance to glyphosate from parental event MON89788. While Bt toxins are expressed in several genetically engineered maize and cotton plant events, MON87701 and its stack MON87701 × MON89788 are the first Bt soybean varieties cultivated in countries, such as Brazil and Argentina, to be given authorisation for import into the EU. This review discusses some specific health risks posed by the genetically engineered soybeans and the risk assessment undertaken by the European Food Safety Authority based on the data from the company Monsanto, which carried out field trials in the US and Argentina [1].

Monsanto, however, did not investigate any combinatorial health effects emerging from the stacked trait. The data provided by the applicant concern acute toxicity testing with a high dosage of the isolated Bt toxin in mice [2]. In addition, they provided two 90-day studies with meal derived from MON87701; the data from these studies showed a range of uncertainties, such as significant changes in body weight [2]. Monsanto also provided data on allergenicity [1, 2]. Some of the findings are discussed in the relevant passages below.

We elaborate on potential health impacts due to toxic, immunogenic or combinatorial effects involving the Bt toxins, and on the residues from spraying with the complementary herbicide. As yet, there has not been a fully comprehensive review of possible health impacts due to Bt toxins expressed in genetically engineered plants in interaction with constituents from soybeans that, in addition, are resistant (used herein as a synonym for ‘tolerant’) to glyphosate or other herbicides.

### Assessment of the toxicity of Bt toxins

When reviewing existing data, it has to be taken into account that most of the data on the toxicity of Bt toxins are generated by using organisms primarily relevant for environmental risk assessment. But several conclusions derived from these data are also relevant for the health risk assessment of food and feed derived from Bt crops, especially if other specific data are not available or not sufficient. At the same time, the data provided by the applicant do not appear sufficient to draw final conclusions: For example, an acute toxicity study with a high dosage of the isolated Bt toxin in mice [2]. This study does not allow conclusions to be drawn on the toxicity of exposure to the Bt toxin at lower dosages over longer periods of time. Furthermore, combinatorial effects that can emerge from the stacked event were completely left out of risk assessment.

Bt toxins are produced by soil bacteria *Bacillus thuringiensis* [3]. In their native form, a subgroup of Bt toxins, classified as Cry toxins, are mostly regarded as safe for human health and the environment because of their mode of action, that requires a basic pH and some specific receptors and enzymes [4]. The combination of these preconditions are known to occur in the gut of insects, but are absent in other animals such as vertebrates. In genetically engineered soybean MON87701, a specific Bt toxin is expressed, classified as Cry1Ac.

In contrast to native Bt toxins, there are several reasons to assess in more detail the potential toxicity of Bt toxins expressed in genetically engineered plants: It is known that there are several differences in the structure of the Cry toxin expressed in the genetically engineered plants and those used in traditional mixtures [5, 6]. Traditionally, the Bt protoxin has been used for spraying as protoxin and in crystallised (inactivated) form. However, the Cry toxins expressed in the genetically engineered plants are already solubilised and activated. It is known that changes in the structure of the protein can have considerable influence on the toxicity of the Bt proteins [7]. Therefore, the risk assessment taken from traditional Bt toxins used in biological pest control can only be applied to a limited extent to the Bt toxins expressed in plants.

Further, the Cry toxins are expressed by the plants throughout the whole period of vegetation, while the traditional sprays are used in a time-limited and targeted manner, if necessary. The sprayed proteins can be expected to mostly degrade, while Bt toxins expressed in plants will be present in the harvest and—depending on further processing—will also be present in feed and food.

The mode of action of Cry toxins is not fully understood. To some extent it is even a matter of controversial scientific debate [8, 9]. Several authors [10–14] reach different conclusions with regard to the mode of action in target organisms.

The mode of action is highly relevant for the risk assessment of Bt crops, since it is the scientific basis for assuming selectivity: The risk assessment of Bt toxins is mostly based on the concept that specific receptors are needed to bind and activate the toxin in the gut. However, there are several publications calling into question the role of some of the receptors [8, 10, 15]. Kitami et al. [16] show that Bt toxins can bind not only to specific receptors, but to various proteins that do not have many similarities with each other. Gómez et al. [7] summarise that oligomerisation in most cases depends on specific receptors, but at least toxicity in some mutant Bt proteins does not require these. Taken together, the role of some of the receptors in delivering the toxicity in target organisms seems to be well established. However, there is also evidence that Bt proteins can exert toxicity by different modes of action, which are not as yet fully understood [7]. There are also uncertainties around the precise role of multiple putative receptors identified for individual toxins [9].

It is known that in vivo selectivity can differ considerably from the expected selectivity stemming from the classification of Cry proteins, which is based on their structure [17]. Some of the gaps in the current understanding of the mode of action of Bt toxins and their importance for general risk assessment in non-target organisms were pointed out by Lövei et al. [18]: “Even well-studied Cry toxins have an incompletely determined range of toxicity. Although it is clear that Cry1Ab and Cry1Ac are toxic mainly to Lepidopteran species, it is not yet possible to infer toxin specificity from toxin structure, and thus toxin specificity of a Cry toxin is a scientific hypothesis, not a scientific fact. Moreover, truncation and mutagenesis of synthetic toxins might alter their range of toxicity compared with the native toxins”. Some of these uncertainties are summarised by Hilbeck and Otto [19], who come to the conclusion that “there is presently no way of predicting which species may or may not be affected based on the current state of understanding of the proposed modes of action of Cry toxins”.

These findings are very relevant for the health risk assessment of Bt crops, such as *Intacta* soybeans. Certainly, the absence of specific receptors in mammalian species is not sufficient to conclude that Bt proteins are not toxic for them. Potential effects might be subtle and show up only after chronic exposure. Besides the acute toxicity study already mentioned, Monsanto provided two subchronic 90-day studies with meal derived from MON87701 [2]. The data from these studies showed a range of uncertainties such as significant changes in body weight [2]. EFSA did not, however, request further feeding studies over a longer period of time.

Further, no feeding studies with the stacked event were requested [1], and thus potential combinatorial effects remain untested. This means that substantial gaps remain in the risk assessment of the *Intacta* soybeans: Not only is the mode of action of the Cry proteins not fully understood, there are also open questions regarding combinatorial or cumulative effects. There are several publications pointing out that Bt proteins are highly likely to show synergies and interactions with other stressors and plant enzymes (for overview see [20]). For example, it is known that co-stressors, such as cadmium and nematodes, can cause toxicity of Cry toxins in slugs [21, 22], which can be taken as a relevant model organism. These experiments are of general relevance for risk assessment since they show that even organisms that are not known to be susceptible to Cry proteins can be impacted if exposed to other stress factors. It can be hypothesised from these experiments, that co-stressors can render toxicity of Bt toxins independently of the presence of specific known receptors. Thus, selectivity of Bt toxins as expected from experiments with organisms exposed to the Bt toxins alone might not be observed in combination with other stressors.

Multiple challenges or stressors continuously affecting organisms can be regarded as the normal real-life situation in the field as well as in regard to consumption of food and feed. Additive or synergistic effects of Bt toxins in combination with other stressors are also relevant for the health risk assessment of *Intacta* soybeans. Although empirical data on mammals are mostly lacking, the data that are available can be used to derive robust hypotheses for further investigation of health risks: Combinatorial effects are known to enhance the toxicity of Bt toxins in invertebrates and have been observed in experiments with pyrethroids [23], azadirachtin, [24], avidin [25], bacteria [14], nosema [26] and other Bt toxins [27, 28]. Since the stacked *Intacta* soybeans and food and feed derived thereof are likely to contain residues from spraying with glyphosate formulations, the possible interaction between Bt toxins and co-stressors, such as pesticides, are relevant to the health risk assessment.

Other examples of synergies that are specifically relevant for the health risk assessment of *Intacta* soybeans include the effect that protease inhibitors can have on the toxicity of Bt toxins: Such an inhibition could delay the digestion of proteins, including Cry toxins, and thus enhance toxicity by prolonged or increased exposure. An up to 20-fold increase of toxicity was found even in the presence of very low levels of protease inhibitors [29–31]. Soybeans are known to show high levels of such inhibitors e.g. the trypsin-inhibitor [32]. The degradation of these proteinase inhibitors in the soybeans will depend on the method of heat processing. There are several methods, such as micronisation, roasting, expanding, extrusion or hydrothermal processing, that all work with different temperatures and durations [33]. Germination is used, for example, to produce soymilk [34]. The methods used will depend on the product to be placed on the market [35] as well as on the variety used [32]. The degradation of the inhibiting proteins will vary, but they will not be removed completely [32].

Several combinatorial mechanisms that can enhance the toxicity of Bt toxins have thus far been described; these are also relevant for the risk assessment of plants expressing these toxins. However, none of these mechanisms were discussed by EFSA when assessing *Intacta* or its parental plants. Table 1 gives an overview of some of the relevant combinatorial effects of Bt toxins. While some combinatorial effects might only occur under some circumstances, protease inhibitors are abundant in soybeans, and therefore need to be taken into account in the risk assessment of Bt soybeans. Furthermore, residues from spraying with herbicide formulas based on glyphosate are particularly relevant as additional stressors, since these residues can be expected to be present in most soybeans harvested from *Intacta*.

**Table 1:** Overview of some combinatorial effects of Bt toxins known to enhance toxicity

Description of combinatorial effects known to enhance toxicity of Bt toxins	Some references
Availability of specific co-factors	Broderick et al. [12]
Parallel or successive exposure to biotic and/or abiotic stressors	Kramarz et al. [21], Kramarz et al. [22], Khalique and Ahmed [23], Singh et al. [24], Zhu et al. [25], Mason et al. [14], Reardon et al. [26]
Delaying degradation of Bt toxins by plant enzymes (protease inhibitors)	Zhang et al. [29], Zhu et al. [30], Pardo Lopez et al. [31]
Other Bt toxins	Sharma et al. [27], Tabashnik et al. [28]

As yet, most of the findings on combinatorial effects stem from organisms that are relevant for environmental risk assessment, but these are also relevant for health risks. In addition, there are findings in mammalian species showing that Bt toxicity is a relevant topic for detailed health risk assessment: Some Cry toxins are known to bind to epithelial cells in the intestines of mice [36, 37]. As far as potential effects on health are concerned, several authors [38–43] show that Cry proteins could potentially have an impact on the health of mammals. Also de Souza Freire et al. [44] confirm haematoxicity of several Cry toxins. Some of these effects seem to occur where there are high concentrations and tend to become stronger after several days. Such observations highlight the need to study the effects after long-term exposure to lower dosages and/or in combination with relevant herbicides as described above.

### Assessement of immune system responses

Effects of Bt toxins on the immune system have been identified in different species and via different routes, including whole food dietary administration. The observations include studies on mice [45, 46], and pigs [47, 48]. Immune system responses have also been shown for fish [49, 50]. Based on data that were provided to the Indian authorities by Monsanto, Gallagher [51] also assumed immunotoxic reactions in rats: Rats fed with Bt aubergine-producing Cry1Ac protein were significantly less healthy than controls as demonstrated by an increased white blood cell count, eosinophils in particular, and enlarged spleens. Further hepatotoxic effects included elevated bilirubin and acetylcholinesterase.

Rubio-Infante and Moreno-Fierros [52] summarised some findings regarding the Cry1Ac toxin and its effect on the immune system. They classify Cry1Ac as a potent mucosal and systemic immunogen and adjuvant [36, 53]. They mention the high immunogenicity of the Cry1Ac protoxin demonstrated by its capacity to induce significant specific antibody responses in serum and mucosal-secretions recovered from the small and large intestine, bronchoalveolar and vaginal lavages of mice after immunisation by every tested route, such as intraperitoneal, intragastric, intranasal, rectal [36, 54] and vaginal [55]. In summary, the adjuvant effects of Cry1Ac protoxin were evaluated regarding the specific antibody responses attained at both mucosal and systemic levels to co-administered antigens of different nature. In a further publication [56], it is shown in more detail how Cry1Ac induces macrophage activation. Rubio-Infante and Moreno-Fierros [52] conclude further risk research is necessary: “(…) the immunogenicity of these proteins and their possible risks in humans after short- and long-term exposure must be determined. Evaluation of the risks of Cry proteins in other systems such as the respiratory and nervous systems is also needed. The toxicity definition must include the adverse effects caused by these toxins not only in the short term; therefore, subchronic and chronic studies in humans should be performed, and the immunotoxicological features of these toxins should be determined”.

The relevance of these questions for the risk assessment of food and feed is evident: Immune system responses can have various impacts on health, especially under permanent or long-term exposure. For example, the University of Manchester [57] identified several diseases, such as Coeliac disease, food protein-induced enterocolitis and food protein-induced enteropathies, that can be associated with non-IgE-mediated immune adverse reactions to foods.

In particular, Cry1Ac which is expressed in *Intacta* soybeans is known to also act as an adjuvant that can boost immune reactions to antigens [52]; thus the Bt toxin can for example enhance the reaction of the immune system to allergens being present in the diet. This is particularly relevant in the case of Bt soybeans, since soybeans (i.e. some of their proteins) belong to the group of food plants categorised as known human allergens [58, 59].

As far as the MON87701 modified soybean is concerned, the only empirical investigation on immune system responses to this soybean provided by the applicant was carried out with 13 samples from sera from patients with known allergic reaction to soybeans [60, 61]. In the case of MON89788, it was restricted to 16 such samples [62, 63]. For MON87701, the outcome was unclear, there were some differences when comparing the samples with the controls, which were difficult to interpret. Although submitted as regulatory documents, none of the studies met the Good Laboratory Practice (GLP) quality standards [60–62]. In addition, Rice et al. [63] did not make any statement on GLP. No empirical testing was performed with the stacked event.

The investigations were carried out with a very low number of samples. It is surprising therefore that EFSA accepted these studies: As the minutes of a meeting of the working group (WG) “Self Task on Allergenicity” from 24 September 2007 show, experts from EFSA had serious doubts about the reliability of investigations with sera from patients with known allergic reaction to soybeans as performed in this case. According to the minutes [64], “More sera from patients are needed but they also need to be well-characterised. Statistical calculations have been done showing that 60–70 well-characterised sera are needed based on variability. Since this might not be feasible, the WG has to consider the reliability of studies with a lower number of sera”.

Further EFSA guidance [65] requires specific investigations to exclude risks for children and elderly people if risks concerning the immune system have to be assessed: “The specific risk of potential allergenicity of GM products in infants as well as individuals with impaired digestive functions (e.g. elderly people, or individuals on antacid medications) should be considered, taking into account the different digestive physiology and sensitivity towards allergens in this subpopulation”. However, these specific risks for infants and other relevant groups were left aside during EFSA risk assessment for *Intacta* and its parental plants [1, 2].

Besides the test with sera from patients, potential allergenicity in parental plants was assessed by applying a pepsin digestion assay. As a result, the Cry protein is thought to degrade rapidly in the gastrointestinal tract and the risk of triggering immune system responses was regarded as being low. However, Chowdhury et al. [66] and Walsh et al. [47] have found Cry1Ab proteins in the lower part of the gastrointestinal tract of pigs fed with a diet containing Bt maize. This directly challenges the above-mentioned view of rapid degradation of Cry1A proteins in the stomach. It appears that the Cry1A proteins can show a much higher stability in monogastric species than predicted by current in vitro digestion experiments. Further, and more specifically in regard to *Intacta* soybeans, it has to be taken into account that the Cry protein expressed in the soybeans might be much more resistant to degradation than that expressed in maize, due to the occurrence of a higher level of proteinase inhibitors. Thus, Bt toxins are not likely to be degraded rapidly in the gut and can persist in larger amounts until digestion is complete and there is enough time for interaction between various food compounds. The need for further investigations was also confirmed by Guimaraes et al. [67], who showed that Cry1Ab proteins were stable and conserved their immunoreactivity by using a physiologically more relevant digestion model. In addition, a study commissioned by EFSA [68] shows that the in vitro pepsin tests used to date are not likely to provide reliable results.

To summarise some of the findings, for the soybean *Intacta* and its parental plant MON87701, there are two factors that indicate a higher risk to the immune system in comparison to other plants such as Bt maize and Bt cotton. These two factors should be used for further hypothesis-driven research. Firstly, soybeans produce protease inhibitors that can prolong exposure to Bt proteins during digestion and therefore increase the likelihood of immune system responses. Secondly, soybeans produce many plant allergens, and there is a specific risk that the Bt protein can enhance the immune system response to these compounds at the consumption stage.

In assessing these questions, it should also be taken into account that Bt toxins from other transgenic plants, such as stacked maize, can be mixed with the soybeans in the diet and thereby enhance immune system responses based on the mechanisms identified, even if the soybeans do not express Bt toxins themselves. For example, genetically engineered plants, such as the genetically engineered maize ‘Smartstax’ (MON89034 × 1507 × MON88017 × 59122), express up to six Bt toxins, resulting in a much higher concentration of the potentially immunogenic proteins. We are not aware of studies that have investigated the impact on the immune system of consuming food or feed derived from these plants alone or in combination with soybeans.

### Assessing the Bt concentration

Interaction with environmental stressors and extreme weather conditions relevant in times of climate change, such as drought, can also impact the Bt concentration in the plants [69]. Huge variations in Bt concentration have, for instance, been found in genetically engineered cotton plants [70]. As known from other cases—such as MON810 (a genetically engineered maize expressing Cry1Ab Bt toxin), independent research [71] has shown that the data provided by industry do not show the true range of variation of Bt toxins in the plants. Trtikova et al. [72] show that the stress reactions of maize MON810 are not predictable. In the case of the genetically engineered soybeans MON87701 and the stacked event *Intacta*, there have been no independent and systematic investigations to determine the Bt concentration in varying environmental conditions. As a result, the true range of variation of Bt concentration in the plants is not fully known.

Furthermore, in comparison to the parental plants, the stacked soybean *Intacta* tends towards a higher concentration of the newly expressed proteins in its tissues as well as a higher degree of standard deviation [73]. This indicates emerging genomic effects in the stacked soybeans or other interactions that cannot necessarily be predicted from the parental plants. The relevant data available in this regard (see Table 2) are derived from a small number of samples and from only one season.

**Table 2:** Summary of Cry1Ac protein concentration in soybean leaf tissues collected from MON87701 × MON89788 in comparison to MON87701 grown in Argentina during 2007–2008 [73]

Tissue type	Mean (SD) Range (µg/g fwt)	Mean (SD) Range (µg/g fwt)
	MON 87701 × MON 89788	MON 87701
OSL-1	90 (37) 50–190	90 (17) 68–120
OSL-2	60 (21) 22–96	53 (25) 34–130
OSL-3	38 (21) 12–90	27 (9.6) 11–45
OSL-4	63 (26) 32–110	31 (8.2) 13–47

Number of samples per OSL: 14 for MON87701 and 15 for MON87701 × MON89034 (see also list of abbreviations)

In general, fully evaluated protocols able to deliver reproducible and comparable results are needed to determine the true range of variation of Bt concentration in the plants and the expression rate of the newly introduced proteins. It is known that slight differences in the method/protocol used in measuring using ELISA can lead to huge differences in the results [6]. However, no validated method has as yet been made available to independent laboratories for Cry1Ac expressed in the soybeans, with the result that major uncertainties remain about the exact concentration of Bt toxin expressed in the plants.

There is a further requirement for data relating to the effect processing has on the Bt proteins under a sufficiently broad range of technical conditions. As far as the effects of processing on the derived products are concerned, the soybeans have only been subjected to one specific kind of heat processing, chosen by Monsanto without any clear justification [74]. As Bell et al. [74] state: “The temperature (~190 °C) and duration (~15 min) used in this assessment were selected to represent a baking treatment that might be employed in the production of foods that contain soybean flour”.

As already mentioned, there are several methods, such as micronisation, roasting, expanding, extrusion, hydrothermal processing and germination, that all work with different temperatures and durations. Germination is a method used, for example, to produce soymilk [33]. The methods used will depend on the product to be placed on the market [34] as well as on the variety of soybean [35]. The degradation of the inhibiting proteins will vary, but they will not be removed completely [35]. Most of these standard methods work with temperatures much lower than 190 °C. In general, each processing company might also prefer to vary the standardised methods, since the goal of the processing is not only to degrade anti-nutritional compounds, such as trypsin-inhibitors, but also to produce a food or feed product with high quality proteins and healthy compounds, such as isoflavones [34]. If the methods used particularly focus on the conservation of protein quality in the soybeans, this could result in the structure and function of the Bt toxin being preserved in food and feed.

In general, the method as described by Bell [74] cannot be considered to be sufficient to assess the effect of processing on the concentration of Bt proteins in the plant. Very limited conclusions can be drawn on the factual exposure of consumers and animals if the soy is used in food and feed because there are no data on the effects from any other technical treatments used to process soybeans. Since the authorisation is not limited to specific purposes, usage as soybean sprouts or soymilk may serve as examples where technical treatment is highly limited. Such products could reach the market without any further risk assessment.

To summarise, based on the current data, it is not possible to determine exposure to Bt toxins within the food and feed chain, although this would be directly relevant for the assessment of risks to the immune system as well as for other potential effects. The assumptions presented by the applicants concerning exposure of livestock and humans are not based on data derived from sufficiently reliable methods. Further, the data presented do not show the true range of variations of Bt toxins in the plants grown under various environmental conditions.

It has to be stressed that these questions not only concern genetically engineered soybeans that express Bt toxins. And in assessing these questions, it should also be taken into account that Bt toxins from other transgenic plants, such as stacked maize, can be mixed with the soybeans in the diet and enhance immune system reactions based on the identified mechanisms.

### Assessment of residues from spraying

Since *Intacta* soybeans not only produce an insecticidal toxin but are also tolerant to glyphosate, the question arises for the health risk assessment about specific residues from spraying and potential interactions with the Bt toxins.

In general, the risk assessment of genetically engineered herbicide-resistant plants currently performed by EFSA is divided into the assessment of the plant performed by the GMO-panel, and assessment of the pesticide performed by the pesticide panel. However, this does not mean that if a pesticide is authorised for use in the EU, that no further investigation of the residues from spraying with the complementary herbicide is needed. Due to the specific agricultural practices that go along with the cultivation of these herbicide-resistant plants, there are, for example, specific patterns of applications, exposure and occurrence of specific metabolites and an emergence of combinatorial effects that require special attention. For example, large-scale commercial cultivation of these plants results in a strong selective pressure on weeds to develop resistance to the herbicides [75]. This problem is also relevant for health risk assessment, since this has led to increasing amounts of glyphosate being sprayed [76] and subsequently more residues in the harvest [77]. Furthermore, herbicide-tolerant plants are engineered to survive the application of the complementary herbicide, while most other plants will die after a short time. Thus, for example, residues of glyphosate, its metabolites and additives to the formulated product might accumulate and interact in the plants, also changing the plant’s composition [78]. Finally, in stacked events, such as *Intacta*, a combination of specific plant constituents is present in the genetically engineered plants. The combination of the residues from spraying and of insecticidal proteins (as in the case of *Intacta*) leads to a unique pattern of combinatorial exposure in the feed and food chain.

As a publication by Kleter et al. [79] shows, using herbicides to spray genetically engineered herbicide-resistant plants does indeed lead to patterns of residues and exposure that are not taken into account in regular pesticide registration: “1. GM herbicide-resistant crops can change the way that herbicides can be used on these crops, for example: (a) post-emergent over-the-top applications (i.e. on the crop itself) instead of directed sprays, avoiding herbicide contact with the crop; or (b) pre-emergent and pre-harvest applications made to the conventional crop and not, or in different quantities, to the GM crop. 2. The residue profile of the applied pesticide may have been altered on the basis of the nature of the modification. 3. The overall pattern of pesticides applied to the particular crop may have been altered, leading to different exposure to pesticide residues overall”.

Thus, according to a reasoned legal opinion drawn up by Kraemer [80], from a regulatory point of view, residues from spraying with complementary herbicides have to be taken into account in the risk assessment of genetically engineered plants.

The EU pesticide regulation also requires specific risk assessment of imported plants if the usage of pesticides is different in the exporting countries compared to countries in the EU: Recital 26 of Regulation 396/2005 requires Maximum Residues Levels (MRLs) to be set for food and feed produced outside the Community if produced by different agricultural practices as regards the use of plant protection products. Article 14 of Regulation 396/2005 requires that the presence of pesticide residues arising from sources other than current plant protection uses and their known cumulative and synergistic effects are determined. Further, Article 29 of Regulation 1107/2009 states that active substances and synergists have to be approved, and the maximum residue levels for each specific agricultural product have to be determined.

Whatever the case may be, both the EU pesticide regulation and the GMO Regulation require a high level of protection for health and the environment. Thus, in regard to herbicide-resistant plants, the specific assessment of residues from spraying with complementary herbicides must be considered to be a prerequisite for granting authorisation. In addition, additive or synergistic effects need to be investigated if a plant contains or produces other compounds with potential toxicity.

Consequently, authorisation for the import and usage in food and feed of genetically engineered plants cannot be granted if the plants contain residues from spraying with complementary herbicides that pose unacceptable risks, or are suspected of being harmful to the health of humans and/or animals.

A basic prerequisite for risk assessment in this context is the availability of valid and reliable data on residue loads from spraying with herbicides. This is especially relevant in the case of glyphosate: A study published in 2015 by IARC [81] claimed that glyphosate is probably carcinogenic to humans. While carcinogenicity of the active ingredient remains a matter of debate [82], it is documented that additives and their mixtures used in the commercial formulations for spraying glyphosate show a much higher toxicity than the active ingredient alone [83].

The amount of residues depends on the specific agronomic management used in the cultivation of the herbicide-resistant plants. Data from publications show [77, 78] a considerable amount of residues from spraying can be expected in genetically engineered soybeans that are resistant to glyphosate formulations. In general, the level of residues is likely to increase due to increasing problems with herbicide-resistant weeds [76]. However, there is a lack of reliable data covering the actual range of residue load in the plants [79]. EFSA also stated in 2015 [84], that the safety of residues from spraying glyphosate formulations could not be concluded on the data provided by the company. Thus, EFSA was unable to deliver a conclusive risk assessment on the actual risks of residues from spraying with glyphosate and the various glyphosate formulations used in the countries where such plants are cultivated. Therefore, the data regarding residues from spraying glyphosate on the genetically engineered soybeans do not meet the requirements of Regulation 1107/2009 or Regulation 396/2005. However, neither the EU risk assessor nor the EU risk manager discussed this aspect.

In conclusion, the residues from spraying are inevitable constituents of the plant composition, leading to a specific pattern of exposure via food and feed that needs to be addressed during the authorisation process of genetically engineered herbicide-resistant plants.

Furthermore, in the context of the risk assessment of the stacked event *Intacta*, the residues from spraying might interact with the Bt toxin and might act as a potent co-stressor. Thus, the combinatorial effects between the effects of glyphosate and the Bt toxins need to be assessed in more detail. For example, Kramarz et al. [21] show interaction with co-stressors can render toxicity of Bt proteins to organisms that are not susceptible to Bt toxins alone. In addition, Bøhn et al. [85] show that additive effects of several Cry toxins and Cry toxins interact with Roundup/glyphosate when co-exposed. However, these aspects were not discussed during EU risk assessment.

In addition, there are some other issues related to residues from spraying with glyphosate that should be considered during the health risk assessment of *Intacta* soybeans. Two examples are mentioned here briefly:

There is a considerable amount of literature indicating that glyphosate formulations can act as so-called endocrine disruptors (see, for example, [86–89]). Since soybeans also produce a number of plant oestrogens with hormonal activity [90], there might be some synergistic or additive interaction with the residues from spraying with glyphosate formulations. However, potential impacts from the consumption of products derived from soybean MON89788 and *Intacta* on the hormonal system of mammals were not investigated.

A further example is the potential impact on the intestinal microbiome. Such effects might be caused by the residues from spraying, since glyphosate was shown to have negative effects on the composition of the intestinal flora of cattle [91] and poultry [92]. In addition, Walsh et al. [93] also describe effects on the microbiota in pigs fed with Bt maize MON810. They consider these changes to be beneficial but point out that more investigations would be needed. No data on the potential impacts of *Intacta* soybeans on the intestinal microbiome have been published so far.

## Conclusions

In regard to assessment of health risks, the case of *Intacta* highlights several regulatory problems with Bt soybean plants in the EU: (1) There are potential combinatorial effects between plant components and other impact factors that might enhance toxicity—this could also be relevant for the assessment of herbicide residues in food and should, therefore, be investigated in more detail. (2) It is known that Bt toxins have immunogenic properties; since many allergens occur naturally in soybeans, these immunogenic properties raise specific questions regarding health effects that so far have not been taken into account during risk assessment. (3) Fully evaluated and reliable protocols are needed to determine the Bt concentration in the plants, in addition to a comprehensive set of data on gene expression under varying environmental conditions. Further, detailed data on the effects of processing and intestinal degradation are needed to assess the exposure of the food chain to Bt toxins. Neither protocols nor detailed data were requested during the process of authorisation for *Intacta* and its parental plants. (4) The permanent exposure to residues from spraying with herbicides in combination with a mixture of Bt toxins in the food chain requires specific approaches for risk assessment.

It remains a matter of discussion whether the standards currently applied by EFSA meet the requirements for risk assessment as defined in EU regulations such as 1829/2003 and Directive 2001/18. In general, the concept of comparative risk assessment needs some major revision. Priority should be given to more targeted approaches, such as the investigation of the plant reactions to environmental stressors, risks to the immune and the hormonal system of mammalian species, combinatorial effects and long-term impact assessment of permanent consumption. Further, it does not make sense that stacked events showing a higher degree of complexity due to possible interactions should undergo a lower level of risk assessment than the parental plants.

In regard to the risk manager, the EU Commission, the current practice of authorisation does not appear to comply with EU regulation requirements, such as 1829/2003 and Directive 2001/18, that obligate a high level of protection for human health and the environment taking into account the precautionary principle as the underlying paradigm.

Some of the issues, such as the assessment of toxicity, immunogenic effects and the assessment of residues from complementary herbicides, are under the remit of the risk assessor, i.e. the European Food Safety Authority (EFSA). And some issues will require closer collaboration between the risk manager, i.e. the EU Commission and EFSA, including requesting industry to deliver protocols for measuring the Bt concentration in the plants and to close the gaps between pesticide regulation and GMO regulation.

Since further soybean varieties expressing Bt toxins are already in the pipeline of companies, such as Monsanto, these issues require urgent attention. Further, many of the issues raised also concern other genetically engineered plants that express insecticidal proteins or are engineered to be resistant to herbicides, or are being combined in stacked events.

## Abbreviations

EFSA: European Food Safety Authority
Bt: *Bacillus thuringiensis*
OSL: over-season leaf tissue from for stages of vegetation
SD: standard deviation
fwt: fresh weight
