# Erratum to: Possible health impacts of Bt toxins and residues from spraying with complementary herbicides in genetically engineered soybeans and risk assessment as performed by the European Food Safety Authority EFSA

**DOI:** 10.1186/s12302-017-0107-z

**Published:** 2017-02-22

**Authors:** Christoph Then, Andreas Bauer‑Panskus

**Affiliations:** Testbiotech, Institute for Independent Impact Assessment in Biotechnology, Frohschammerstr. 14, 80807 Munich, Germany

## Erratum to: Environ Sci Eur (2017) 29:1 DOI 10.1186/s12302-016-0099-0

Upon publication of the original article [1], the authors noticed that the number of the event mentioned in the first sentence of the article was incorrect. Instead of: MON89788, the correct number is: MON87701 and therefore the first sentence of the article should read:

“MON87701 was the first genetically engineered soybean worldwide to express a Bt toxin.”
